# Role of stem cells in ocular diseases: Progress and challenges

**DOI:** 10.7555/JBR.39.20250251

**Published:** 2026-05-21

**Authors:** Tongyao Fu, Yan Ma, Yifei Jiang, Chang Jiang, Xiumiao Li, Qin Jiang

**Affiliations:** 1 The Affiliated Eye Hospital, Nanjing Medical University, Nanjing, Jiangsu 210029, China; 2 The Fourth School of Clinical Medicine, Nanjing Medical University, Nanjing, Jiangsu 210029, China

**Keywords:** stem cells, cell therapies, ocular diseases, progress, challenges

## Abstract

Ocular diseases, including corneal disease, glaucoma, age-related macular degeneration (AMD), diabetic retinopathy, and retinopathy of prematurity, can significantly impair vision and reduce quality of life. Because degenerated cells in these diseases are unable to regenerate, treatments for these conditions have limited efficacy. Stem cell therapies are revolutionizing the treatment of degenerative eye conditions, enabling structural and functional restoration through mechanisms such as cell replacement and paracrine signaling. This review examines advances in stem cell therapy for ocular diseases, from preclinical studies to early clinical trials, focusing on various types of stem cells, including embryonic stem cells, induced pluripotent stem cells (iPSCs), and mesenchymal stem cells. Significant progress has been made with iPSC-derived retinal pigment epithelial cell transplantation in AMD treatment, showing cell survival in trials, and with mesenchymal stem cells for corneal repair through anti-inflammatory effects. Challenges remain, such as controlling differentiation to prevent tumorigenesis, managing immune rejection, and ensuring manufacturing processes that comply with Good Manufacturing Practice standards. By integrating mechanistic insights with translational strategies, this review outlines pathways to optimize stem cell therapies for previously intractable ocular diseases.

## Introduction

The eye is a crucial sensory organ in the human body, and a variety of ocular diseases, including corneal disease, glaucoma, age-related macular degeneration (AMD), diabetic retinopathy (DR), and retinopathy of prematurity (ROP), can significantly impair vision and reduce quality of life^[1]^. These diseases all involve cellular degeneration, and most of the affected cells cannot regenerate^[2–3]^. Currently, therapeutic approaches mainly focus on symptomatic treatments through medication, laser therapy, and surgery. However, the effectiveness of these treatments is limited, highlighting the urgent need for innovative approaches to promote cell regeneration.

Pluripotent stem cells, renowned for their ability to differentiate into organoids^[4]^, and adult stem cells, which can differentiate into specific tissue cell types^[5]^, hold great promise for vision restoration and tissue repair^[6–8]^. Currently, corneal epithelial stem cells^[9–10]^ and mesenchymal stem cells (MSCs) are among the most successfully applied stem cell types used in clinical applications^[11–12]^. For instance, limbal stem cells offer a potential treatment for corneal diseases such as limbal stem cell deficiency (LSCD). Retinal disorders may also benefit from therapies using human embryonic stem cells (ESCs) or retinal pigment epithelial (RPE) cells derived from induced pluripotent stem cells (iPSCs)^[8,13–16]^.

Despite advancements in the use of stem cells in ophthalmology, several challenges remain. These include issues with sourcing and transplantation techniques^[17]^, as well as concerns about safety^[18]^, efficacy, and variability in the quality and quantity of stem cell studies across different ocular tissues^[19]^. This article reviews recent progress in stem cell research for ocular diseases and emphasizes the need to further explore underlying mechanisms, optimize therapeutic strategies, and advance clinical applications of stem cells in ophthalmology^[20]^.

## Stem cells

Stem cells are undifferentiated cells with remarkable abilities to self-renew and regenerate functional tissues. They are present in both embryos and adult organisms, and are classified based on their differentiation potential, origin, and lineage. Depending on their potency, stem cells can be categorized as totipotent, pluripotent, multipotent, oligopotent, or unipotent^[21]^. They can also be classified by source into ESCs, iPSCs, and somatic stem cells. ESCs and iPSCs exemplify pluripotency^[22]^, while somatic multipotent stem cells include hematopoietic stem cells (HSCs)^[23]^, MSCs^[24]^, neural stem cells (NSCs)^[25]^, and endothelial stem/progenitor cells (EPCs)^[26]^. Their biological characteristics encompass self-renewal, multilineage differentiation potential, plasticity, repair capacity, epigenetic regulation, migration, and homing ability.

**Figure 1:**
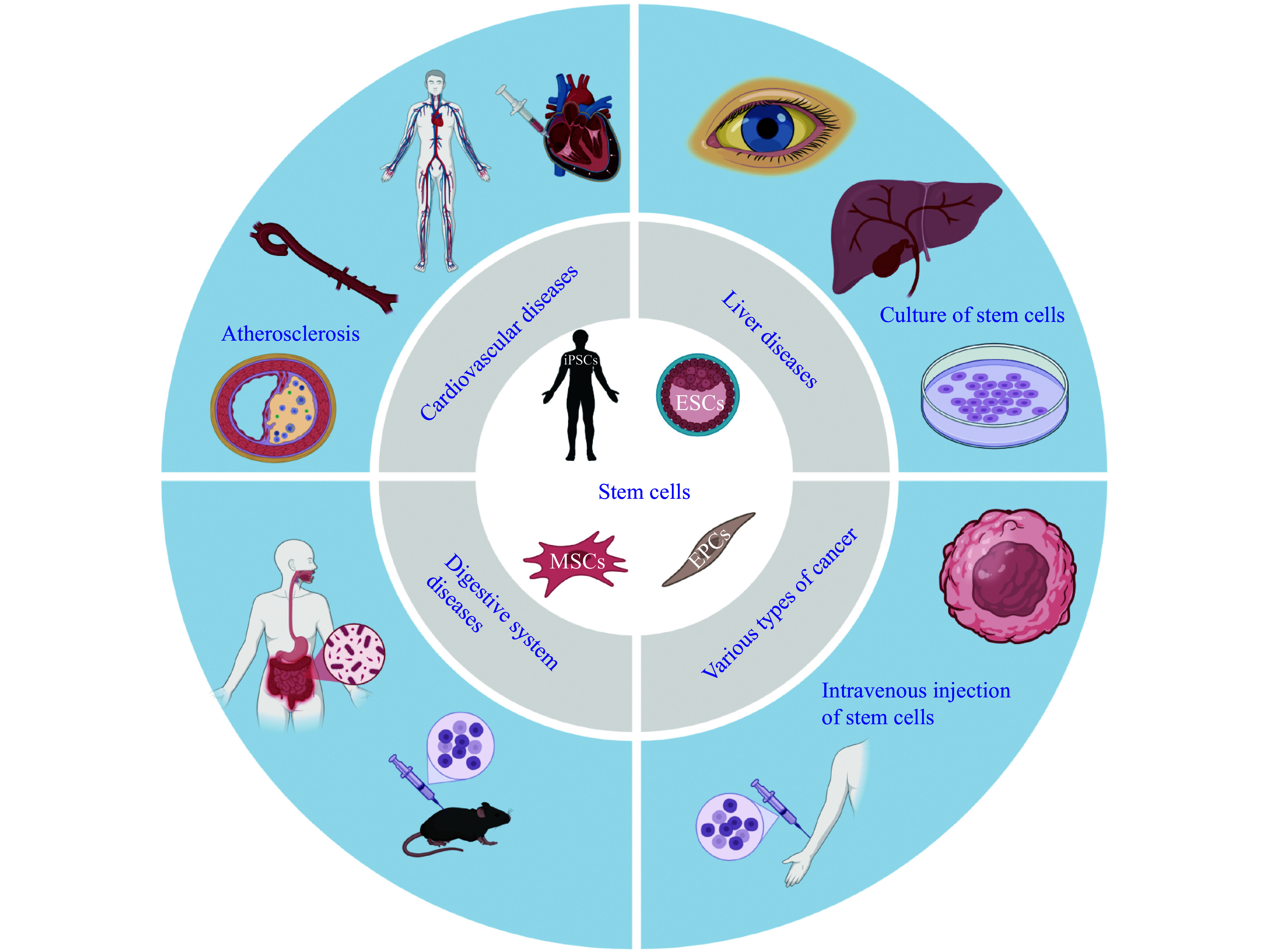
Illustration of the applications of stem cell therapy across a wide range of clinical settings. Stem cell therapy is being applied across a wide range of clinical settings, such as cardiovascular diseases, digestive system diseases, liver diseases, and various types of cancer. Abbreviations: ESCs, embryonic stem cells; MSCs, mesenchymal stem cells.

Stem cell therapy utilizes these regenerative properties to treat various conditions by repairing or replacing damaged tissues with healthy cells^[5]^. This process can involve the use of autologous stem cells—derived from the patient's own body—or allogeneic stem cells sourced from donors^[27]^. In recent years, stem cell therapy has been applied in a wide range of clinical settings, including cardiovascular diseases^[28]^, digestive system diseases^[29]^, liver diseases^[30]^, and various types of cancer^[31]^ (***Fig. 1***).

**Figure 2:**
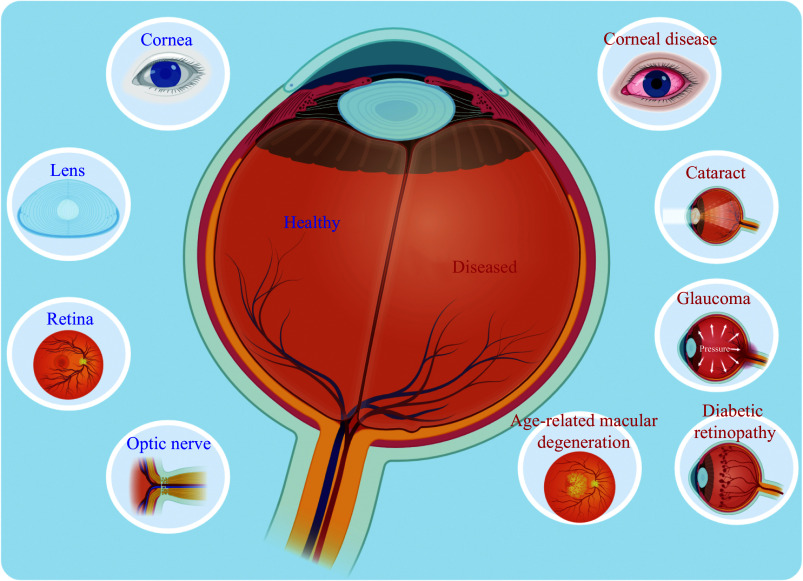
Ocular stem cell therapy in health and disease. Schematic representation of ocular anatomy and ocular diseases targeted by regenerative therapies.

The human eye, a complex organ formed through the coordination of neuroectodermal, ectodermal, and mesodermal tissues^[32]^, can suffer impairments that lead to blindness. Blinding ocular diseases represent a significant global health challenge in the 21st century. By 2020, an estimated 43 million people worldwide were blind, with another 295 million experiencing moderate to severe vision loss^[33]^. Ocular regenerative therapies hold great promise for transforming the treatment of conditions such as corneal disease, cataracts, glaucoma, AMD, DR, and retinitis pigmentosa (RP)^[19]^ (***Fig. 2***).

We believe that the eye represents a promising pioneering field for the application of stem cell therapy, particularly in advanced diseases characterized by cell loss. Moreover, the potential integration of stem cell and gene therapies presents an exciting prospect that could revolutionize the treatment landscape for these conditions^[19]^.

## Ocular disease with stem cells

### Cornea: repair of three layers

The cornea functions as the window to the visual system, serving as both a protective barrier and a lens that focuses incoming light. Damage to corneal integrity and transparency can cause visual impairment and affect over 23 million people worldwide^[34]^. Untreated corneal defects can lead to corneal blindness, the second most prevalent form of blindness after cataracts. In severe cases, corneal transplantation becomes necessary, although the risk of graft rejection can be as high as 90% in high-risk patients due to innate and adaptive immune responses^[35–36]^. Stem cell-based therapies have shown promise in restoring function to the three primary corneal layers: the superficial epithelium, the stroma, and the endothelium (***Table 1***).

**Table 1:** Stem cells for the treatment of corneal diseases

Target diseases	Stem cells	*In vitro* model & findings	*In vivo* model & findings	Refs.
Sterile corneal injury	Bone marrow mesenchymal stem cells	N/A	Acute corneal alkali burns in rats; subconjunctival injections; accelerated corneal wound healing, attenuated inflammatory responses, and reduced neovascularization; decreased CD68^+^ cells and downregulated macrophage inflammatory protein-1α, tumor necrosis factor-α, and vascular endothelial growth factor.	[53]
Sjögren's syndrome-associated dry eye disease	Human umbilical cord mesenchymal stem cells	Macrophages; activated AKT signaling pathway, triggered activation of M2-related molecules, and upregulated arginase 1 expression.	Rabbit with Sjögren's syndrome-associated dry eye disease; systemic infusion; attenuated chronic inflammation.	[56]
Keratitis	Human lipid-derived bone marrow stromal cells	N/A	Mice with corneal wounds; prevented the formation of light-scattering scar tissue; blocked neutrophil infiltration by secreting tumor necrosis factor-α stimulated gene 6.	[61]
Keratitis	Corneal stromal stem cells	N/A	Mice with liquid nitrogen-induced corneal stromal scarring; anterior chamber injection; regenerated transparent stromal tissue; exosomal miR-29a and miR-381-5p anti-inflammatory and anti-fibrotic.	[67]
Keratitis	Human embryonic stem cell-derived corneal endothelial cells	N/A	Rabbit with endothelial dysfunction; corneal endothelial cell (CEC)-like cell sheet transplanted surgically; CEC expressed N-cadherin, forkhead box protein C1, and paired-like homeodomain transcription factor 2; restored corneal transparency.	[70]
Keratitis	Human pluripotent stem cell-derived corneal epithelial progenitors	N/A	Rabbit and non-human primate models; CEC-like cell sheet transplanted by surgery; nicotinamide prevented premature senescence and endothelial-mesenchymal transition of transplanted corneal epithelial progenitors.	[71]
Abbreviation: N/A, not applicable.				

#### Corneal epithelial regeneration

The anterior surface of the cornea is covered by a non-keratinized epithelium that continuously renews itself through stem cells located in the basal epithelium of the limbus—a transitional zone between the cornea and the conjunctiva^[37]^.

(1) LSCD

LSCD leads to the loss of corneal transparency and visual impairment^[38]^. LSCD disrupts the limbal barrier function, prompting adjacent conjunctival cells to migrate in an attempt to repair the cornea, a process known as conjunctivalization^[39–40]^. This results in loss of visual clarity^[41]^, as well as inflammation, chronic pain, photophobia, and severe visual impairment^[40,42]^ due to changes in the ocular surface environment. Conjunctival stem cells possess bipotency, allowing them to proliferate into both conjunctival epithelial cells and other epithelial cell types, supporting conjunctival regeneration^[43–45]^. While allogeneic limbal transplantation has been used to treat LSCD, results are often inconsistent due to postoperative complications such as infectious keratitis or immune rejection^[46–47]^. Oie *et al*^[48]^ used autologous cultured limbal epithelial cell sheets in clinical trials for patients with unilateral LSCD and reported promising outcomes: two years after surgery, 70% of patients exhibited corneal epithelial regrowth, and 60% experienced improved visual acuity. Additionally, an innovative culture system has been developed to transform oral mucosal epithelial cells into corneal epithelial tissue equivalents, providing a new approach for treating LSCD^[49]^.

(2) Sterile corneal injury

Abnormal immune responses are considered a primary factor in the development of many ocular surface disorders, including inflammatory and degenerative corneal conditions. Among these, sterile injuries to the cornea are a leading cause of vision loss worldwide^[50]^. Oh *et al*^[51]^ emphasized the beneficial effects of MSCs in treating such injuries, noting their anti-inflammatory, pro-regenerative, anti-apoptotic, and anti-angiogenic properties.

Bone marrow stem cells (BMSCs) are frequently used in regenerative medicine, because they can be sourced as autologous grafts or from donors, making them ideal candidates for human clinical applications^[52]^. Yao *et al*^[53]^ investigated the effects of subconjunctival MSC injections on healing acute corneal alkali burns in rats. Their results indicated that these injections significantly accelerated corneal wound healing, reduced inflammatory responses, and decreased neovascularization in alkali-burned corneas. The underlying mechanisms were attributed to a reduction in CD68^+^ cells and a downregulation of pro-inflammatory cytokines, such as macrophage inflammatory protein-1α (MIP-1α), tumor necrosis factor (TNF-α), and vascular endothelial growth factor (VEGF)^[53]^. In another promising development, self-erecting ectodermal autonomous multiregional ocular cells, derived from human iPSCs (hiPSCs), have been successfully used to restore corneal function in animal models of corneal blindness^[54]^. Furthermore, MSCs derived from bone marrow, adipose tissue, and the corneal stroma have shown regenerative potential in various sterile corneal injury models involving mice, rats, and rabbits^[55]^. These findings suggest that MSCs protect the corneal epithelium by modulating the immune response and stimulating the regeneration of corneal epithelial cells.

(3) Sjögren's syndrome-associated dry eye disease (SS-DED)

SS-DED is an autoimmune disease characterized by the progressive infiltration of lymphocytes into the lacrimal gland (LG) and ocular surface. This process results in a type of dry eye known as aqueous-deficient dry eye and ocular surface epithelial defects^[51]^. Recent studies have shown that systemic administration of human umbilical cord MSCs (hUC-MSCs) effectively attenuated chronic inflammation and improved clinical symptoms in rabbit LGs affected by SS-DED. Mechanistically, hUC-MSCs activated the AKT signaling pathway within macrophages, which subsequently triggered the activation of M2-related molecules and an upregulation of Arg1 expression^[56]^. In a recent clinical trial, Møller-Hansen and colleagues evaluated the safety and feasibility of administering allogeneic adipose-derived MSCs into the LG. Seven participants with aqueous-deficient dry eye received a single transconjunctival injection of these MSCs into the LG of one eye. Following this treatment, significant improvements were observed: tear osmolarity decreased, tear film break-up time increased, and tear production improved as measured by Schirmer scores. Additionally, there was a trend toward reduced corneal staining. Importantly, these benefits were sustained for up to 16 weeks^[57]^.

#### Corneal stromal regeneration

The transparency of the cornea largely depends on its stroma, particularly on the precise arrangement of collagen fibrils. Corneal keratocytes are essential for maintaining both corneal transparency and shape by secreting and degrading the extracellular matrix (ECM)^[58]^. However, when injured or infected, these stromal cells can form persistent, opaque scar tissue^[19]^. Currently, the primary treatment for corneal damage involves corneal transplantation, either lamellar or penetrating keratoplasty, which presents challenges such as tissue rejection and a shortage of donor tissues^[59]^. Stem cell therapy has emerged as an exciting alternative to keratoplasty.

MSCs can be expanded from small, clinically reproducible corneal limbal biopsy samples obtained from the scleral limbus of human cadaveric corneas. These human limbal basal stem cells can differentiate into specific corneal cell types that express characteristic marker genes, such as *ALDH3A1*, *AQP1*, *KERA*, and *PTGDS*, and produce well-formed collagen and keratan sulfate polysaccharides. When transplanted into mouse corneal wounds, limbal basal stem cells prevent the formation of light-scattering scar tissue, which typically contains fibrous stromal components, indicating their potential for autologous stem cell-based treatments aimed at corneal stromal blindness^[60]^. The mechanism behind scar prevention in mice involves MSCs inhibiting neutrophil infiltration into the cornea by secreting the protein tumor necrosis factor alpha-induced protein 6 (TSG-6)^[61]^.

Since the discovery of corneal stromal stem cells (CSSCs) in 2005, stem cell therapy has emerged as a promising strategy for preventing or repairing corneal scarring^[62–63]^. CSSCs are clonal, genetically stable, and multipotent, capable of differentiating into various functional cell types, including corneal keratocytes, and thus show considerable promise for corneal regeneration^[62]^. Even after approximately 12 years of cryopreservation, CSSCs maintain their stem cell properties, including labeling capacity and differentiation potential. These cells can be isolated from both donor eyes and patients through a small corneal limbal biopsy without compromising corneal function, making them an ideal autologous source for corneal regeneration^[64–65]^. In research conducted by Ghoubay *et al*^[66]^, liquid nitrogen was used to induce stromal scarring in mice, characterized by initial inflammation, keratocyte apoptosis, conversion of keratocytes into myofibroblasts, type Ⅲ collagen production, impaired stromal ultrastructure, corneal opacity, increased stiffness, and reduced visual acuity. This study demonstrated that CSSCs could facilitate the regeneration of transparent stromal tissue after such induced scarring. Mechanistically, Yam *et al*^[67]^ described the anti-inflammatory and anti-fibrotic effects of miR-29a and miR-381-5p in CSSCs, which contributed to scar prevention. The expression of miR-29a in extracellular vesicles (EVs) distinguished CSSCs with anti-scar properties.

#### Corneal endothelial regeneration

The innermost layer of the cornea, known as the corneal endothelium, is crucial for maintaining corneal hydration and transparency due to its barrier and fluid transport functions. It can be damaged by endothelial dystrophy or surgical trauma^[68]^. One of the major challenges in regenerative medicine is the limited capacity of human corneal endothelial cells (CECs) to proliferate, coupled with the difficulty in effectively delivering these cells to the posterior region of the cornea^[68]^. Despite these obstacles, the straightforward structure, accessibility, and absence of blood vessels in the cornea make it an ideal candidate for pioneering stem cell-based regenerative therapies^[19]^. In one study by Shao *et al*^[69]^, researchers investigated the potential of transforming bone marrow-derived endothelial progenitor cells (BEPCs) into CECs to repair corneal endothelial defects. They used a scaffold constructed from porcine corneal acellular matrix and transplanted the induced BEPCs into feline corneas lacking Descemet's membrane and endothelium. The transformed BEPCs closely resembled CECs, displaying polygonal shapes and expressing markers such as aquaporin-1 as well as tight cell junctions. By 28 days post-surgery, the treated corneas began to regain transparency. In another study, Zhang *et al*^[70]^ developed a method to generate CEC-like cells from human ESCs (hESCs) by progressing through the peripapillary mesenchymal precursor stage. These CEC-like cells expressed markers like N-cadherin and transcription factors forkhead box C1 (FOXC1) and paired-like homeodomain 2 (PITX2). When transplanted into a rabbit model of endothelial dysfunction, these cells gradually restored corneal transparency. More recently, Li *et al*^[71]^ introduced an innovative therapy combining human pluripotent stem cell (hPSC)-derived corneal endothelial progenitor cells (CEPs) with nicotinamide. This approach successfully restored corneal clarity and thickness in models using rabbits and non-human primates. Nicotinamide prevented premature aging and unwanted transdifferentiation of transplanted CEPs into other cell types within the transforming growth factor-beta (TGF-β)-rich environment of the eye, ensuring their maturation into functional CECs *in vivo*.

### Retinal degenerative diseases

Retinal degenerative diseases are among the primary causes of currently incurable blindness worldwide^[72–73]^. The leading causes of irreversible blindness are neurodegenerative diseases, which include conditions resulting from the death of retinal ganglion cells (RGCs), photoreceptors, and the associated RPE, as well as those caused by the loss of the RPE itself, such as glaucoma, optic nerve disease, AMD, and RP^[74]^. The prospect of regenerating neural tissue to reverse blindness in eye diseases is promising, and various approaches have been proposed to achieve this, including replacing lost cells or restoring cell function in damaged tissue. Consequently, regenerative medicine, particularly cellular therapy for retinal degenerative conditions, offers substantial promise^[75]^ (***Table 2***).

**Table 2:** Stem cells for the treatment of retinal degenerative diseases

Target diseases	Stem cells	*In vitro* model & findings	*In vivo* model & findings	Refs.
Glaucoma	Human trabecular meshwork stem cells (hTMSCs)	N/A	Mouse model induced by laser-damaged trabecular meshwork (TM); anterior chamber injection; hTMSCs homed to TM and prevented inflammation and fibrosis by C-X-C chemokine receptor type 4/stromal cell-derived factor 1 chemokine axis.	[80]
Glaucoma	hTMSCs	N/A	Mouse; anterior chamber injection; hTMSCs homed to TM and differentiated into TM cells expressing chitinase-3-like protein 1.	[82]

#### Glaucoma and optic nerve disease

Glaucoma is a chronic neuropathy characterized by structural changes at the optic nerve head and the progressive loss of RGCs. It is the leading cause of irreversible blindness worldwide, with projections suggesting that its prevalence will exceed 100 million individuals by 2040^[76]^. Current clinical management targets the reduction of intraocular pressure (IOP), the only modifiable risk factor for glaucoma, by decreasing aqueous humor production or enhancing outflow *via* alternative pathways such as the uveoscleral route^[77]^. However, some patients continue to experience disease progression even after achieving the lowest safely attainable IOP. The trabecular meshwork (TM) is integral to maintaining the normal function of the conventional aqueous outflow pathway^[78]^. Research using glaucoma mouse models has shown that TM cell death is correlated with increased IOP, underscoring its importance in fluid regulation^[79]^. Coulon *et al*^[78]^ demonstrated that the repair of TM cells improved aqueous outflow, prevented RGC apoptosis, and preserved vision despite elevated IOP.

(1) Trabecular meshwork restoration in glaucoma and ocular hypertension

Research on TM stem cells (TMSCs) has been conducted by various groups^[80]^. In 1989, a study observed that after laser trabeculoplasty in human post-mortem eyes, certain TM cells migrated approximately six times their original distance to a region anterior to the filtering segment of the meshwork^[81]^. This migration provided evidence for the presence and repopulating potential of resident stem cells within the TM.

Du *et al*^[82]^ explored the capability of human TMSCs to home to and persist in mouse TM tissue when transplanted *in vivo*. They introduced DiO-labeled TMSCs into the anterior chamber of normal mice, where these cells primarily settled in the trabecular meshwork, maintaining their presence for at least four months. This investigation demonstrated that stem cells extracted from human TM and cultured *in vitro* have the capacity to localize and differentiate within the TM environment. These findings suggest potential applications for developing innovative cell-based treatments for glaucoma. Yun *et al*^[80]^ also validated the homing ability of TMSCs in a mouse model induced by laser photocoagulation. Their results indicated that TMSCs preferentially migrated and integrated into the laser-damaged TM region. The transplantation of TMSCs prevented laser-induced inflammation and fibrosis while restoring structural integrity and function. Mechanistically, the CXCR4/SDF-1 chemokine axis played a crucial role in directing TMSC homing. Xiong *et al*^[83]^ used a mouse model of glaucoma featuring a transgenic myocilin Y437H mutation. In this model, the transplantation of TMSCs into the TM significantly reduced IOP and protected RGCs. Mechanistically, these TMSCs enhanced TM cell viability, facilitated the release of myofibrillar proteins into the aqueous humor to alleviate endoplasmic reticulum stress, and repaired TM tissue by modulating the extracellular matrix and ultrastructure.

A study employing a laser-induced open-angle glaucoma model evaluated the potential role of MSCs in tissue repair mechanisms. The injection of MSCs into the anterior chamber resulted in the regeneration of the TM and a reduction in IOP. The researchers hypothesized that this regenerative effect was facilitated by secreted factors from the MSCs and the activation of intrinsic repair processes, rather than through direct integration with host tissue, which was found to be temporary (lasting only four days)^[84]^. Another study by Roubeix *et al*^[85]^ showed that MSCs regulated IOP by increasing TM cell viability, decreasing TM contractility, or inducing phenotypic changes in TM cells. Specifically, when hTM cells were exposed to TGF-β2 in conjunction with MSC-cultured medium, the latter decreased phosphorylation of myosin fibers and altered expression levels of specific ECM components. These findings suggest that MSCs can reduce TM contractility and influence phenotypic transitions.

In a study, Zhu *et al*^[86]^ transplanted mouse iPSC-derived TM (iPSC-TM) cells into the anterior chamber of 6-month-old transgenic mice expressing a pathogenic variant of human myocilin (MYOC^Y437H^). IOP and aqueous humor outflow facility were recorded for a period of three months. Twelve weeks after transplantation, the number of endogenous TM cells significantly increased. Morphologically, transplantation of iPSC-TM cells maintained the ER structure of TM cells, as observed through transmission electron microscopy. Another study investigated whether transplanting iPSC-TM cells could restore TM cellularity and function in elderly donor eyes. hiPSCs were differentiated into iPSC-TM cells, and the cell density of these TM cells significantly increased in eyes receiving iPSC-TM injections at both seven and 14 days post-transplantation. The results indicated that iPSC-TM transplantation stimulated the proliferation of endogenous TM cells in perfusion-cultured aged human donor eyes, suggesting that functional TM regeneration is achievable in eyes affected by primary open-angle glaucoma^[87]^ (***Table 3***).

**Table 3:** Advantages and disadvantages of TMSCs, MSCs, and iPSCs in glaucoma

Stem cells	Advantages	Disadvantages
TMSCs	1. As resident stem cells in the trabecular meshwork, they have intrinsic tissue compatibility; 2. Strong homing ability: can specifically colonize the trabecular meshwork and survive for a long time (at least four months); 3. Directly participate in the homing and regeneration of the trabecular meshwork.	1. Limited sources: need to be isolated from autologous or allogeneic trabecular meshwork tissue. 2. Culture and expansion *in vitro* may be restricted by cell senescence.
MSCs	1. Wide sources (*e.g.*, bone marrow, umbilical cord) and easy to obtain and culture. 2. Exert repair effects through paracrine signaling (*e.g.*, activating intrinsic repair mechanisms) instead of relying on direct integration with host tissues.	1. Weak direct integration ability: survival time in the host tissues is short (only four days). 2. Dependent on secreted factors for function, with potentially weaker effects compared with cells that directly participate in regeneration.
iPSCs	1. Unrestricted sources (can be induced from autologous somatic cells), avoiding immune rejection. 2. Can be differentiated into a large number of functional trabecular meshwork-like cells, suitable for long-term repair.	1. Complex differentiation process: strict induction into trabecular meshwork-like cells is required; otherwise, functional abnormalities may occur. 2. Long-term safety (*e.g.*, tumorigenicity) needs further verification.
Abbreviations: iPSCs, induced pluripotent stem cells; MSCs, mesenchymal stem cells; TMSCs, trabecular meshwork stem cells.		

(2) Transplantation of RGCs

Optic neuropathies are a group of diseases that affect RGC axons in the optic nerve, often leading to permanent vision loss. Glaucomatous optic neuropathy is a primary global cause of irreversible blindness. In diseases such as glaucoma, RGCs degenerate and do not regenerate in adult mammals. Stem cells are used in the therapeutic approach for glaucoma and optic neuropathies to protect existing cells from further damage^[19]^.

In one study, GFP-labeled RGCs were transplanted into rat retinas by intravitreal injection. The transplanted RGCs exhibited morphological characteristics similar to those of endogenous RGCs, with axons projecting to the optic nerve head in the host retina and dendrites extending toward the inner plexiform layer. These axons eventually reached brain regions, including the lateral geniculate nucleus and the superior colliculus. Electrophysiological recordings from the transplanted RGCs indicated that their electrical excitability and photoresponses were comparable to those of the host's ON, ON–OFF, and OFF RGC subtypes, albeit slower and more adaptable. These findings offer a promising approach for the development of cell replacement strategies to address RGC degeneration in diseased retinas^[88]^. Fetal human retinal progenitor cells (hRPCs), currently undergoing preclinical trials and preparation for regulatory approval, demonstrate potential for neuroprotection and may hold significant value in treating glaucoma^[89]^. Wang *et al*^[90]^ injected primary-cultured hRPCs into the vitreous cavity of RCS rats. After four weeks of follow-up, electroretinograms (ERGs) showed that b-wave amplitude was significantly higher in hRPCs-treated groups than in the untreated group; protective effects were maintained for up to eight weeks. Mechanistically, hRPCs released several neurotrophic factors, including GDF-15, PDGF-AA, EGF, and NT-4, which show strong potential for reversing RGC degeneration. Recently, investigators devised a rapid protocol for direct differentiation of RGCs from human stem cells, utilizing NGN2 overexpression. These data demonstrate that iRGCs can be rapidly generated *in vitro*, similar to human fetal RGCs. Furthermore, iRGCs transplanted intravitreally survived and migrated into the mouse retinal tissue, thereby protecting host RGCs from neurodegeneration. Thus, the simplicity of the system could facilitate translational research on human RGCs^[91]^.

A study demonstrated that the transplantation of human umbilical cord blood stem cells (hUCBSCs) exerted therapeutic effects in rats with experimental optic neuropathy. After intravitreal transplantation of hUCBSCs, RGC survival was significantly increased. Mechanistically, this was correlated with the continuous secretion of brain-derived neurotrophic factor (BDNF) and glial-derived neurotrophic factor (GDNF) by hUCBSCs *in vivo*^[92]^. Similarly, researchers co-cultured physically isolated porcine neural retina and human MSCs to evaluate the paracrine neuroprotective effects of MSCs on neural retina degeneration. In this *in vitro* model, the results showed that MSCs delayed retinal glial cell degeneration, which was correlated with increased levels of secreted neurotrophic factors, such as BDNF and ciliary neurotrophic factor (CNTF)^[93]^. Recently, in a large clinical study known as the Stem Cell Ophthalmology Treatment Study (SCOTS), six patients with dominant optic atrophy were enrolled. They were subsequently treated with a combination of retrobulbar, subtenon, intravitreal, or subretinal BMSC placement, followed by an intravenous injection of autologous BMSCs. Binocular vision improved in 83.3% of the patients. Mechanistically, it is hypothesized that mitochondrial translocation and neuroprotective exosomes secreted by BMSCs may play a key role in ameliorating this mitochondrial disease^[94]^.

#### Age-related macular degeneration and RPE transplantation

AMD is the leading cause of vision loss among the elderly in developed countries^[95]^. This disease is typically categorized into two types: neovascular and non-neovascular subtypes. The neovascular type is characterized by the presence of type 1, 2, or 3 macular neovascularization^[96]^. The non-neovascular type is typically associated with advanced geographic atrophy. The loss of RPE, as observed in the atrophic (dry) form of macular degeneration, leads to photoreceptor dysfunction and subsequent degeneration, ultimately resulting in blindness^[74]^.

In one study, 12 participants with advanced Stargardt disease, a leading cause of macular degeneration in children and young adults, received subretinal transplantation of up to 200000 hESC-derived RPE cells, accompanied by 13 weeks of systemic immunosuppressive therapy. No significant efficacy was reported, although borderline improvements in visual acuity were detected in four out of 12 participants^[97]^. Another study by Mandai *et al*^[16]^ involved the transplantation of autologous iPSC-derived RPE cell sheets into the subretinal space of patients with neovascular AMD. iPSCs generated from skin fibroblasts of two advanced neovascular AMD patients were differentiated into RPE cells. One year after surgery, the transplanted cell sheets remained intact, showing neither improvement nor deterioration in the best-corrected visual acuity, and cystoid macular edema persisted. A phase 1/2a clinical trial examined the long-term effects of scaffold-based implants of human embryonic stem cell-derived RPE cells in patients with advanced geographic atrophy. After three years of follow-up, no subretinal implant migration was observed in the nine patients with dry AMD. Visual acuity improved by five letters in BCVA in eyes with retinal implants compared with those without implants^[98]^. In a separate study, Saini *et al*^[99]^ documented the generation of hiPSCs from AMD patients, including two donors with a rare homozygous ARMS2/HTRA1 genotype. hiPSC-derived RPE cells expressed multiple AMD/drusen-associated proteins, and those from AMD donors exhibited substantially elevated levels of complement or inflammatory factors. In another study, porcine cell-free scleral or uveal ECM or corresponding hydrogels were developed for the delivery of hiPSC-derived RPE cells (hiPSC-RPE). Results indicated that ECM-hydrogel-delivered hiPSC-RPE cells rescued photoreceptor cell death and retinal glial cell lesions, restoring visual function in rats with retinal degeneration over the long term^[100]^.

We are now at an exciting juncture, with several stem cell-based trials underway around the globe. The stimulation of endogenous adult RPE cells to generate new autologous RPE cells without transplantation surgery^[101]^ is a promising future direction.

#### Retinitis pigmentosa and photoreceptor transplantation

Retinal photoreceptor cells are specialized neuronal cells responsible for detecting light. These include rods, which are activated in low-light conditions, and cones, which are triggered by bright light of specific wavelengths. Cones are densely concentrated in the macula, the central area of the retina, which is crucial for high-acuity vision. Once lost, photoreceptors, as terminally differentiated neurons, cannot regenerate or be replaced^[19,102]^. RP is a genetically and clinically diverse group of inherited retinal disorders, primarily characterized by rod photoreceptor degeneration, followed by cone degeneration and eventual atrophy of the RPE. RP is the leading cause of inherited blindness, with a global prevalence of about one in 3000 to one in 4000 individuals^[103–104]^. Stargardt disease is another inherited retinal condition, a recessive form of macular dystrophy, with an estimated prevalence of about one in 8000 to one in 10000. The disease initiates in the parafoveal region of the macula and progressively involves the foveal region, ultimately resulting in central vision loss and legal blindness. With disease progression, it causes photoreceptor and RPE degeneration, leading to continuous vision loss^[105]^.

Huang *et al*^[106]^ demonstrated that the transplantation of hESC-derived retinal progenitor cells (RPCs) into the subretinal space of RCS rats helped protect Müller cells, preventing gliosis and delaying photoreceptor degeneration, thus preserving retinal function. This effect may be mediated by hERO-RPC-derived small extracellular vesicles (hERO-RPC-sEVs), which influence the fate of Müller cells through the downregulation of the nuclear factor Ⅰ transcription factor B (NFIB) *via* microRNA^[106]^. In a clinical study by Hirami *et al*^[107]^, allogeneic transplantation of iPSC-derived retinal organoid sheets was conducted on two patients with advanced RP (jRCTa050200027). In case 1, the transplanted eye exhibited heightened sensitivity to blue and red light, indicating cone-mediated sensitivity, as measured by full-field stimulus threshold testing at 36 and 52 weeks post-transplantation. The patient's central visual fixation ratio and letter recognition test scores also improved compared with baseline. These findings suggest that the transplantation of allogeneic iPSC-derived retinal organoid sheets holds potential as a therapeutic approach, though further investigation into its safety and efficacy in visual function recovery is needed. Recent advancements in retinal organoids, which are three-dimensional structures derived from human PSCs, have demonstrated that they produce robust, intrinsic light-evoked electrical responses similar to those of adult foveal cone cells. A significant proportion of these laboratory-generated cones exhibit photoresponses and membrane physiology comparable to those of intact primate foveae. These findings strengthen confidence in the potential of retinal organoids for cell replacement therapy using functional human cones, drug testing, and their use *in vitro* models for retinal dystrophy^[108]^.

Gasparini *et al*^[109]^ introduced a human cone-specific reporter hiPSC line for generating retinal organoids. When transplanted into a murine model of cone degeneration, the human cones and CRX+ photoreceptors incorporated extensively, demonstrating strong polarization and development of inner and outer segments. This structural integration, along with subsequent *in vivo* polarization, maturation of human photoreceptors, neuronal plasticity, and the absence of barriers to synaptic connectivity, offers promising evidence supporting the notion that transplanted human photoreceptors can effectively integrate into the remaining outer nuclear layer of patients, providing a potential pathway for functional retinal restoration.

### Retinal vascular diseases

The retinal vasculature is the only neurovascular system that can be directly observed by the human eye, and it can be easily assessed through fundoscopy and multiple imaging modalities. This unique window enables clinicians to diagnose and treat retinopathy, as well as detect systemic diseases such as diabetes or hypertension. DR is the most common retinal vascular disease, followed by retinal vein occlusion and retinal artery occlusion^[110]^. When it comes to cell-based therapy targeting retinal vasculopathy, direct replacement of tissues may prove challenging because multiple types of cells within the retina are involved during the pathogenesis of visual impairment^[111]^. Nevertheless, studies in both preclinical and clinical settings are underway to explore the potential of cell-based therapy for retinal vascular disorders. The majority of these studies focus on adult-derived mesenchymal or progenitor cells, which are regarded as multipotent, although their capacity to differentiate into the various retinal cell types damaged by retinal vascular disease is limited. However, these adult cells have shown the ability to promote tissue regeneration through a local paracrine trophic effect^[112]^ (***Table 4***).

**Table 4:** Stem cells for the treatment of retinal vascular diseases and others (continued)

Target diseases	Stem cells	*In vitro* model & findings	*In vivo* model & findings	Refs.
Cataract	Lens epithelial cells (LECs)	Isolated mammalian LECs and required paired box gene 6 (PAX6) and B-cell-specific Moloney murine leukemia virus integration site 1 (BMI1).	Rabbits, rhesus monkeys, and human infants with cataracts; preserved endogenous LECs and regenerated functional lens.	[138]
Uveitis	iPSC-RPE cells	T cells from patients with active uveitis targeted with iPSC-RPE cells; iPSC-RPE cells inhibited the activation of Th1-type T cells.	N/A	[142]
Uveitis	MSCs	N/A	Model of experimental autoimmune uveitis (EAU); MSCs control ocular autoimmune inflammation.	[144]
Uveitis	MSCs	MSCs reduced T-cell proliferation and Th1 & Th17 cytokine secretion, and enhanced interleukin-10 production.	EAU model in rats; intravenous injection; protected retinal structures and integrity.	[145]
Abbreviations: N/A, not applicable				

#### DR

DR is an ocular complication characterized by the progressive emergence of microvascular dysfunction associated with diabetes mellitus^[113]^. This condition presents a range of clinical manifestations, including inflammation, microaneurysms, vascular damage, and subsequent neovascularization, which leads to mild visual impairments and progresses to severe vision loss or blindness^[114]^. Simultaneously, prolonged hyperglycemia in DR initiates a series of pathological processes, including oxidative stress, endothelial cell dysfunction, and inflammatory responses^[115]^. Moreover, DR affects the retinal microvasculature, causing pathological changes such as basement membrane hypertrophy, pericyte depletion, capillary nonperfusion, and increased vascular permeability^[116]^.

An early study established MSC-derived exosomes as a novel therapeutic vehicle for MSC-based therapies. Researchers evaluated the effects of MSC-derived exosomes from rabbit adipose tissue in a diabetic rabbit model, administering them intravitreally to prevent severe retinal degeneration^[117]^. Furthermore, a clinical trial assessed the safety and feasibility of intravitreal injections of autologous human bone marrow CD34^+^ stem cells as a potential treatment for ischemic and degenerative retinal disorders. CD34^+^ cells were injected into six subjects experiencing irreversible vision loss due to retinal vascular occlusion, inherited or dry AMD, or RP. After six months of follow-up, results showed a favorable safety profile for the cell therapy, without intraocular inflammation or hyperproliferative responses, and no deterioration in BCVA or full-field ERGs^[118]^.

In recent years, investigations into stem cell-derived exosomes have attracted considerable attention, particularly regarding their implications for DR. Song *et al* presented a comprehensive review of stem cell-derived exosomes in the therapy of DR^[119]^. These exosomes function by mitigating oxidative stress within retinal capillary intracellular components^[115]^, alleviating retinal endothelial cell inflammation, and simultaneously inhibiting apoptosis while promoting the proliferation of various cell types, including retinal precursor cells, vascular endothelial cells, neuronal cells, and stromal cells^[119]^. Rong *et al*^[120]^ investigated the therapeutic potency of the clinically graded hESC line-derived MSCs (hESC-MSCs) in db/db mice afflicted with DR. Four weeks post intravenous injection of hESC-MSCs, the damaged retinal ERG functions were augmented, microvascular cell dysfunctions were mitigated, and the levels of peripheral blood pro-inflammatory cytokines and chemokines were modulated. These findings indicate that hESC-MSCs may serve as a promising clinical-grade cellular source for DR therapy^[120]^. Analogously, Sun *et al*^[121]^ elucidated that the intravitreal injection of MSC-derived small EVs (MSC-sEVs) improved the function of retinal microvascular endothelial cells and mitigated cellular apoptosis, inflammation, and angiogenesis in both db/db mice and streptozotocin-induced diabetic rats. Furthermore, researchers genetically engineered MSC-sEVs to have high levels of miR-5068 or miR-10228 expression, thereby enhancing the efficacy of retinal repair. Collectively, these findings underscore the potential of MSC-sEVs, particularly engineered ones, as a therapeutic alternative for DR^[121]^.

Recently, a study employed the docosahexaenoic acid (DHA)-derived mediator 7*S*,14*R*-dihydroxy-4*Z*,8*E*,10*Z*,12*E*,16*Z*,19*Z*-docosahexaenoic acid (7*S*,14*R*-diHDHA), which represents a stereoisomeric form of maresin-1, biosynthesized by leukocytes and their associated enzymes. 7*S*,14*R*-diHDHA induced the secretion of trophic growth factors in MSCs by enhancing the production of hepatocyte growth factor (HGF) and VEGF. Furthermore, 7*S*,14*R*-diHDHA potentiated MSC function by increasing MSC density within the retina of db/db mice, thereby alleviating diabetes-induced pericyte loss^[122]^.

#### ROP

ROP is a neurovascular disorder that affects the retinal visual structures. Excessive exposure to exogenous oxygen is considered to be a major contributor to the onset and severity of ROP. In severe cases, this condition can be sight-threatening and may lead to blindness. Treatment options for ROP include laser ablation and intravitreal injection of anti-VEGF agents^[123]^.

To explore the contribution of HSCs to ischemic retinal revascularization, researchers established an adult mouse model of retinal neovascularization. Their findings suggest that adult HSCs with self-renewal capacity possess functional angioblast activity, allowing clonal differentiation into all hematopoietic cell lineages and into endothelial cells that contribute to retinal revascularization. Recruiting EPCs to the site of ischemic injury is essential for neovascularization^[124]^. In another study, Lin^−^ HSCs were found to contain EPCs capable of forming blood vessels. Intravitreal transplantation of Lin^−^ HSCs into neonatal mice selectively targeted retinal astrocytes, which extensively and persistently integrated into the developing retinal vasculature, thereby rescuing and maintaining normal vessel formation^[125]^.

#### Choroidal neovascularization (CNV)

CNV is characterized by the proliferation of abnormal blood vessels, which can lead to rapid and profound vision loss without anti-VEGF therapy^[126]^. CNV penetrates Bruch's membrane to invade the subretinal space between RPE and photoreceptors, resulting in macular disruption and vision loss^[127]^. Notably, anti-VEGF therapy has proven effective in slowing the progression of the disease and preserving vision. However, due to the recurrence of CNV, many patients require repeated anti-VEGF injections over extended periods^[128]^. Several clinical studies, such as the Comparison of AMD Treatments Trials (CATT)^[129]^ and the VEGF Trap-Eye: Investigation of Efficacy and Safety in Wet AMD (VIEW 1 and VIEW 2)^[130]^, have demonstrated that a substantial proportion of patients with occult CNV, where the CNV has not yet penetrated the RPE, experience a longer disease course and a reduced response to anti-VEGF therapy due to the barrier function of the RPE^[131]^.

In preclinical studies, researchers induced a CNV model in GFP chimeric mice by laser, which led to the recruitment of HSCs to the injury site. As early as one week post-injury, GFP^+^ cells contributed to the functional choroidal vasculature. These findings suggest that recruiting stem cells into the eye may represent a novel therapeutic strategy for CNV^[132]^. In another study, Li *et al*^[133]^ inhibited a rat model of laser-induced CNV by subretinal transplantation of *Fbln5*-overexpressing RPE cells. These cells remained viable for at least four weeks and migrated toward the retinal tissue, resulting in a substantial decrease in the area of leakage.

In a clinical study spanning four years of follow-up, a 77-year-old female diagnosed with polypoidal choroidal vasculopathy received 13 intravitreal injections of anti-VEGF drugs over a 29-month period, during which her vision continued to deteriorate. iPSCs were generated from the patient's skin cells, and RPE cells were differentiated from the iPSC clone. An autologous iPSC-derived RPE sheet measuring 1.3 mm × 3 mm was implanted in the fovea after the removal of substantial fibrovascular tissue that contained the polypoidal choroidal vasculopathy network vessels. After four years of follow-up, no regrowth of exudative neovascular vessels or fibrovascular tissue was observed through fluorescein angiography or spectral-domain optical coherence tomography. Visual acuity remained stable, and no further anti-VEGF injections were required^[134]^. Recently, Zhang *et al*^[135]^ developed an HIF-1α siRNA-loaded poly (lactic-co-glycolic acid)-core/lipid-shell hybrid nanoparticle system, using MSCs as carriers in the targeted therapy of experimental CNV. By exploiting the tropism of MSCs, systemic delivery of these siRNA-loaded engineered MSCs effectively suppressed pathological neovascularization, offering a promising targeted nanomedicine strategy for ocular vascular diseases.

### Others

#### Cataract: lens regeneration mediated by endogenous stem cells

The transparent, biconvex lens located in the anterior region of the eye, along with the cornea, plays a key role in refracting light and adjusting its shape (accommodation) to focus properly^[19]^. In lower vertebrates, lens regeneration is feasible during developmental periods, whereas adult regeneration is restricted to certain urodele amphibians *via* transdifferentiation occurring in corneal or iris tissue^[136]^. However, in humans, lens tissue regrowth following cataract extraction is disorganized and does not result in a functional regenerative lens^[19]^. Cataracts are a major contributor to blindness worldwide and are typically treated through surgical lens extraction followed by the implantation of a synthetic intraocular lens^[137]^.

Utilizing endogenous stem cells for tissue regeneration represents the ultimate objective of regenerative medicine. Researchers have isolated mammalian lens epithelial stem/progenitor cells (LECs) and demonstrated that the transcription factors PAX6 and BMI1 are essential for LEC self-renewal. A surgical technique has been developed for cataract extraction that preserves endogenous LECs, enabling functional lens regeneration in human infants with cataracts^[138]^. Congenital cataracts are the primary cause of treatable childhood blindness globally, affecting approximately four in 10000 newborns^[139]^. A clinical study on infants with bilateral congenital cataracts found that the small capsular opening healed in one month, and transparent lens regeneration began within three months. By eight months, the regenerated lens achieved normal central thickness and accommodation. Although the regenerated lens had some defects, the visual axis remained clear, and visual acuity improved. This innovative pediatric cataract surgery remains in the initial evaluation stages, and further research is needed to address potential complications, such as cataract recurrence due to genetic defects and amblyopia resulting from inappropriate visual stimulation during the regeneration process^[140]^.

Could the regenerative approach be extended to adult cataract surgery? Accelerated lens regeneration and the restoration of accommodation in adults could have significant implications for the presbyopic population.

#### Uveitis

Uveitis refers to a group of heterogeneous ocular inflammatory diseases, which are classified as either infectious or non-infectious. Non-infectious cases are immune-mediated and are further divided into those associated with systemic disease and those that are ocular-restricted. Treatment of infectious uveitis targets the pathogen with topical or systemic agents. Collaborative care between ophthalmologists and internists/rheumatologists optimizes management^[141]^.

**Figure 3:**
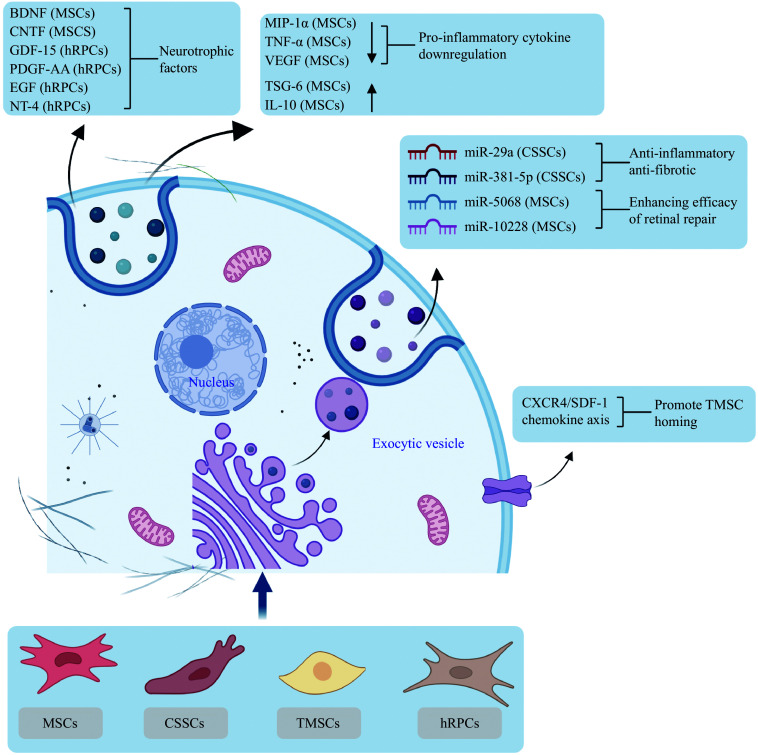
Mechanisms of stem cell therapy. Schematic illustration of mechanisms underlying stem cell therapies, including MSCs, CSSCs, TMSCs, and hRPCs, in ocular tissue regeneration. These cells promote ocular tissue regeneration *via* autocrine/paracrine pathways mediated by neurotrophic factors, cytokines, miRNAs, and chemokine axes (*e.g.*, CXCR4/SDF-1 for TMSCs homing). Abbreviations: BDNF, brain-derived neurotrophic factor; CNTF, ciliary neurotrophic factor; CSSCs, corneal stromal stem cells; CXCR4, C-X-C chemokine receptor-4; EGF, epidermal growth factor; GDF-15, growth differentiation factor-15; hRPCs, human retinal progenitor cells; IL-10, interleukin-10; MIP-1α, macrophage inflammatory protein-1α; MSCs, mesenchymal stem cells; NT-4, neurotrophin-4; PDGF-AA, platelet-derived growth factor-AA; SDF-1, stromal cell-derived factor-1; TMSCs, trabecular meshwork stem cells; TNF-α, tumor necrosis factor-α; TSG-6, tumor necrosis factor-induced protein-6; VEGF, vascular endothelial growth factor.

Recent research has indicated that T cells from patients with active uveitis can be targeted with RPE cells derived from hiPSCs *in vitro*. These iPSC-RPE cells effectively inhibited T-cell activation, leading to a decrease in the expression of interferon-gamma (IFN-γ) and T-bet in CD4^+^ T cells following coculture. The iPSC-RPE cells also significantly inhibited the activation of Th1-type T cells in patients with infectious or non-infectious uveitis, such as HLA-B27-associated acute anterior uveitis, acute retinal necrosis, cytomegalovirus retinitis, sarcoidosis, Vogt-Koyanagi-Harada disease, and Behçet's disease^[142–143]^. The potential of MSCs to control ocular autoimmune inflammation has been demonstrated in an experimental autoimmune uveitis (EAU) model, which serves as a prototype for T-cell-mediated autoimmune diseases of the retina and mirrors autoimmune uveitis in humans^[51,144]^. The therapeutic potential of MSCs was first reported in 2011 by Zhang *et al*^[145]^. In their study, intravenous injection of MSCs (5 × 10^6^ cells per day for three consecutive days) delayed the onset of the disease and reduced its severity, as assessed by clinical and histological scores. MSC treatment reduced T-cell proliferation and the Th1 and Th17 cytokine secretion, while enhancing IL-10 production. Additionally, MSC treatment has exhibited therapeutic and prophylactic effects in recurrent EAU models, where treatment administered following a second episode of EAU significantly reduced severity, protecting retinal structures and maintaining integrity^[146]^ (***Fig. 3***).

### Challenges in delivering ocular regenerative therapy to patients

Stem cell therapies hold great promise for treating previously incurable ocular diseases. Over the last decade, the California Institute for Regenerative Medicine (CIRM)-funded research has focused on several key areas: (a) proving the feasibility of generating transplantable therapeutic cells from stem cells; (b) determining "safe and effective" cell doses through preclinical or clinical studies; (c) exploring optimal cell delivery pathways; (d) ensuring the GMP-compliant production of large quantities of cells for preclinical or clinical studies, as well as potential commercial production; and (e) incorporating new and innovative clinical endpoints into trial designs. The first CIRM-supported clinical trial in ophthalmic diseases demonstrated the feasibility of cell therapy for the retina and vitreous in outpatients. Although long-term data are still limited and the number of patients treated remains small, evidence shows that the implanted cells survive, function, and persist for several months, with encouraging improvements in patients' vision^[147]^.

The use of embryonic, fetal, and adult stem cells in these clinical trials indicates an expanding range of stem cells being evaluated for potential ophthalmic applications. This therapy offers several advantages, including the use of a small number of cells, the simplicity of the procedure, and the ability to directly assess and observe the grafts. However, limitations remain, such as immune rejection of transplanted cells and the tumorigenic potential of stem cells^[148]^. Other challenges involve developing potency assays and ensuring batch-to-batch consistency. In certain cases, specially designed delivery vehicles require development and preclinical testing in large animal models. Whether the eye is truly an immunoprivileged site will require further clinical trials and immunosurveillance to confirm. Key questions include how long immunosuppression should be maintained and whether local immunosuppression can replace systemic immunosuppression^[147]^.

Further research is essential to translate these promising experimental results into clinical applications. This involves developing standardized protocols, optimizing differentiation protocols, ensuring the safety and long-term viability of transplanted cells, and improving delivery methods^[149]^.

## Conclusion and perspectives

Stem cell therapies have made significant strides in the treatment of ocular diseases such as corneal disease, glaucoma, AMD, DR, and ROP, demonstrating their potential to repair damaged tissue and restore vision. Despite this progress, challenges persist. These include difficulties in controlling the directional differentiation of stem cells to ensure accurate differentiation into the desired ocular cell types, improving the efficiency of cell survival and integration after transplantation for better therapeutic outcomes, enhancing management of immune rejection (which is better controlled in some studies), and obtaining long-term safety and efficacy data. Moving forward, it is essential to strengthen multidisciplinary collaboration across biology, medicine, and materials science to overcome technical challenges. Simultaneously, continuous technological innovation should focus on optimizing stem cell culture, differentiation, and transplantation methods, as well as refining clinical trial design and evaluation criteria, thereby promoting the clinical use of stem cell therapies in ophthalmic diseases and bringing hope to more patients.

**Table 2:** Stem cells for the treatment of retinal degenerative diseases (continued)

Target disease	Stem cells	*In vitro* model & findings	*In vivo* model & findings	Refs.
Glaucoma	hTMSCs	TMSCs co-cultured with myocilin mutant TM cells; TMSCs increased the viability of TM cells, reduced endoplasmic reticulum stress, and regulated the extracellular matrix.	Mouse model of glaucoma with a transgenic myosin Y437H mutation; anterior chamber injection; reduced intraocular pressure, increased outflow facility, and protected retinal ganglion cells (RGCs).	[83]
Glaucoma	Mesenchymal stem cells (MSCs)	N/A	Mouse model induced by laser-induced open-angle glaucoma model; anterior chamber injection; reduced intraocular pressure (IOP) by paracrine factors from MSCs, and increased progenitor cell proliferation.	[84]
Glaucoma	MSCs	hTM cells co-applying MSC-cultured medium; hTM cells survive by activating the Akt pathway and decreasing myosin phosphorylation.	Rat model with glaucoma-like ocular hypertension; anterior chamber injection; reduced IOP and protected the death of RGCs.	[85]
Glaucoma	Induced pluripotent stem cell-derived trabecular meshwork cells (iPSC-TM)	N/A	Transgenic mice expressing human myocilin (Tg-MYOCY437H); anterior chamber injection; reduced IOP and restored outflow facility; endogenous TM cells increased and preserved TM cells' endoplasmic reticulum structure.	[86]
Glaucoma	iPSC-TM	hTM cells co-cultured with hiPSC-TM cells; increased hTM cell proliferation.	Human ocular perfusion organ culture system; iPSC-TM stimulated proliferation of endogenous TM cells.	[87]
Optic neuropathies	RGCs	N/A	Rat; intravenous injection; transplanted RGCs acquired the morphology of endogenous RGCs.	[88]
Optic neuropathies	Human retinal progenitor cells (hRPCs)	N/A	Rats; intravenous injection; electroretinography displayed higher b-wave amplitude; hRPCs released growth differentiation factor-15, platelet-derived growth factor-AA, epidermal growth factor, and neurotrophin-4 and restored RGC degeneration.	[90]
Optic neuropathies	RGCs	RGCs generated similarities to human fetal RGCs.	Mice; intravenous injection; protected host RGCs from neurodegeneration.	[91]
Optic neuropathies	Human umbilical cord blood stem cells	N/A	Rats with experimental optic neuropathy; intravenous injection; increased RGC survival by brain-derived neurotrophic factor (BDNF) and glial cell line-derived neurotrophic factor.	[92]
Optic neuropathies	MSCs	Co-cultured neuronal progenitor cells and human MSCs; MSCs delayed retinal glial cell degeneration; increased BDNF and ciliary neurotrophic factor.	N/A	[93]
Age-related macular degeneration (AMD)	Human-induced pluripotent stem cell-derived retinal pigment epithelium (hiPSC-RPE)	N/A	Rats with retinal degeneration; extracellular matrix-hydrogel-delivered hiPSC-RPE cells rescued photoreceptor cell death, retinal glial cell lesions, and restored vision.	[100]
Retinitis pigmentosa	Human embryonic stem cell-derived retinal organoid retinal progenitor cells (hERO-RPCs)	Müller cells; inhibited gliosis and promoted early dedifferentiation.	Rats; subretinal injection; protected Müller cells, prevented gliosis, and delayed photoreceptor degeneration; hERO-RPCs-EVs miRNA-mediated downregulation of nuclear factor I/B.	[106]
Retinitis pigmentosa	Human pluripotent stem cells (hPSCs)	Three-dimensional retinal organoids derived from hPSCs produced robust, intrinsic light-evoked electrical responses.	N/A	[108]
Retinitis pigmentosa	hiPSCs	N/A	Mice model of cone degeneration; subretinal injection of iPSC-derived human photoreceptors promoted polarization and maturation of human photoreceptors.	[109]
Abbreviation: N/A, not applicable.				

**Table 4:** Stem cells for the treatment of retinal vascular diseases and others

Target diseases	Stem cells	*In vitro* model & findings	*In vivo* model & findings	Refs.
Diabetic retinopathy (DR)	Mesenchymal stem cell-derived extracellular vesicles (MSC-EVs)	N/A	Diabetic rabbit model; intravenous injection; prevented severe retinal degeneration; MSC-EV miRNA-222-mediated retinal repair.	[117]
DR	Human embryonic stem cell-derived mesenchymal stem cells (hESC-MSCs)	N/A	Db/db mice afflicted with DR; intravenous injection; augmented retinal electroretinography functions, mitigated microvascular cell dysfunctions, and modulated peripheral blood and retinal inflammation.	[120]
DR	MSC-EVs	Retinal microvascular endothelial cells (RMECs); MSC-EVs alleviated RMEC apoptosis, inflammation, and angiogenesis, resulting in the peroxisome proliferator-activated receptor-γ coactivator-1α (PGC-1α) through enhancer of zeste homolog 2 (EZH2)-induced methylation modification.	Db/db mice and streptozotocin-induced diabetic rats; intravitreal injection; MSC-sEVs improved retinal function; the presence of miR-5068 and miR-10228 in MSC-sEVs targeted the HIF-1α/EZH2/PGC-1α pathway.	[121]
DR	MSCs	MSCs treated with 7S,14R-diHDHA; increased the production of vascular endothelial growth factor and hepatocyte growth factor.	Db/db mice; intravitreal injection; 7S,14R-diHDHA potentiated MSC function by elevating MSC density and alleviating diabetes-induced pericyte loss.	[122]
Retinopathy of prematurity (ROP)	Hematopoietic stem cells (HSCs)	N/A	Mice with retinal neovascularization; self-renewing adult HSCs possess functional angioblast activity; differentiate into endothelial cells that contribute to revascularizing the retina.	[124]
ROP	Endothelial progenitor cell-enriched HSCs	N/A	Mice; intravitreal injection; Lin (−) HSCs targeted retinal astrocytes, rescued and maintained normal vessel formation.	[125]
Choroidal neovascularization (CNV)	HSCs	N/A	The CNV model in green fluorescent protein chimeric mice by laser induction; recruited HSCs to the injury site; GFP^+^ cells contributed to the functional choroidal vasculature.	[132]
CNV	Retinal pigment epithelial cells	N/A	Rats inhibited laser-induced CNV; subretinal injection of fibulin-5 (FBLN5)-overexpressed retinal pigment epithelium (RPE) cells; cells survived and migrated toward the retina; and reduced the leakage.	[133]
CNV	MSCs	N/A	Mice with the CNV model; intravenous injection; MSCs carried by HIF-1α siRNA-loaded nanoparticles; reduced CNV area and length, and inhibited ocular angiogenesis.	[135]
